# Supplementary material for: Early Detection of Hyperprogressive Disease in Non-Small Cell Lung Cancer by Monitoring of Systemic T Cell Dynamics
Source: Cancers (Basel). 2020 Feb 4;12(2):344. doi: 10.3390/cancers12020344 (PMC7073153; doi:10.3390/cancers12020344)
Supplement: Supplementary file 1 [file cancers-12-00344-s001.pdf]

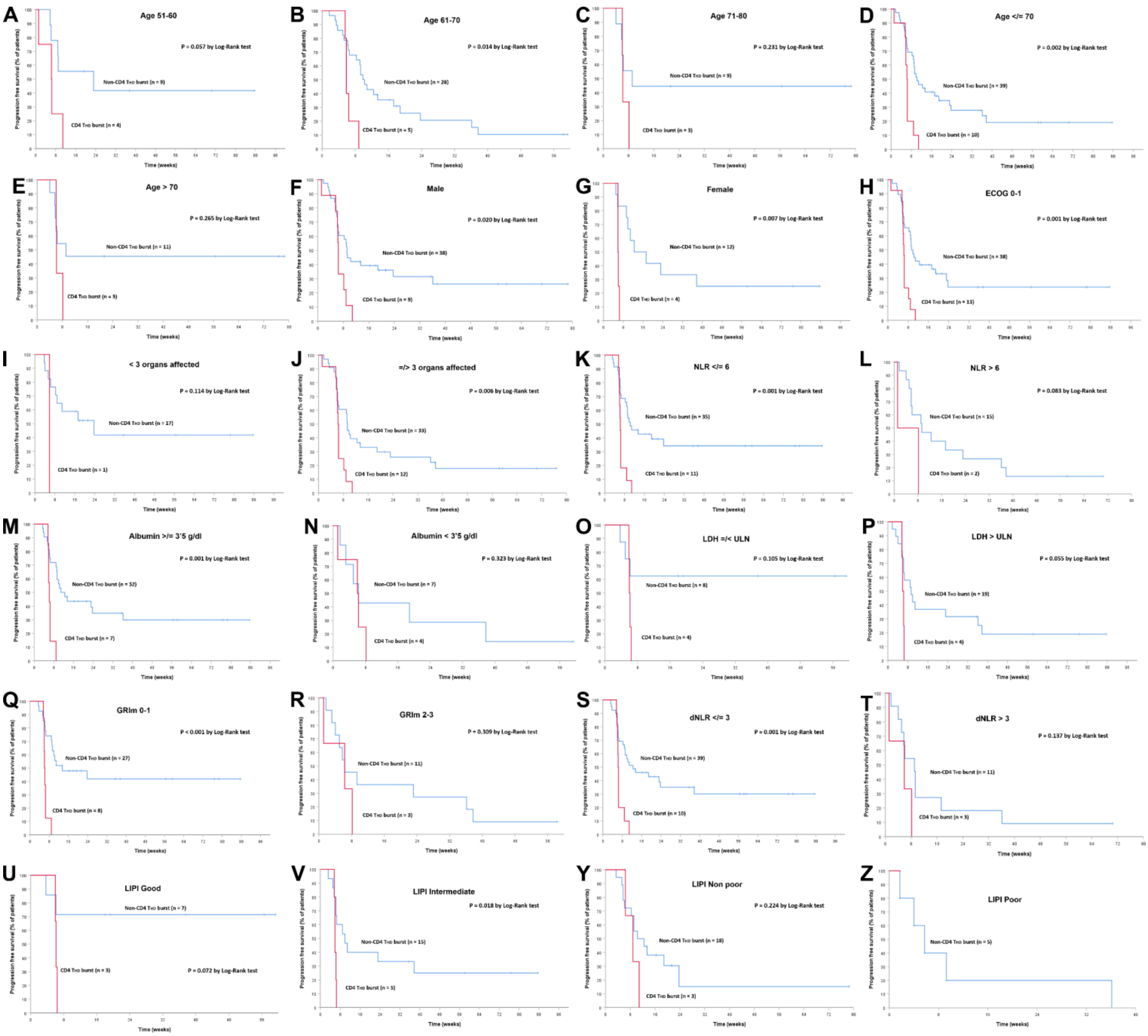

Suplementary Figure. Kaplan-Meier plot for PFS representing immunotherapy-treated patients classified as indicated and, stratified by the incidence of CD4 THD burst (≥ 1.3). (A, B, C, D, E) According to age; (F,G) gender; (H, I, J) ECOG and affected organs; (K, L) neutrophil-to-lymphocyte ratio; (M, N) serum albumin; (O, P) serum LDH; (Q, R) GRim score; (S, T) derived neutrophil-to-lymphocyte ratio; (U, V, Y Z) LIPI.
